# Respiratory syncytial virus genotypes circulating in urban Ghana: february to november 2006

**DOI:** 10.11604/pamj.2014.19.128.4749

**Published:** 2014-10-06

**Authors:** Evangeline Obodai, Richard Asmah, Isaac Boamah, Bamenla Goka, John Kofi Odoom, Theophilus Adiku

**Affiliations:** 1 Department of Virology, Noguchi Memorial Institute for Medical Research, University of Ghana, Legon, Accra, Ghana; 2 Department of Microbiology, University of Ghana Medical School, Accra, Ghana; 3 School of Allied Health Sciences, College of Health Sciences, Korle-Bu, Accra, Ghana; 4 Department of Child Health, Korle Bu Teaching Hospital, Accra, Ghana

**Keywords:** Respiratory syncytial viruses, lower respiratory tract infections, multiplex RT-PCR

## Abstract

**Introduction:**

Respiratory syncytial virus (RSV) is the major cause of acute lower respiratory tract infection (ALRI) in young children. RSV strains have been divided into 2 major antigenic groups (A and B), which are further divided into several genotypes, but very little is known about its circulating genotypes in Ghana. This study characterized RSV genotypes detected in children with ALRI in Accra between February and November 2006.

**Methods:**

Nasopharyngeal aspirates (NPA) were obtained from children diagnosed with ALRI between February and November 2006. The NPA were screened for RSV using a nested multiplex reverse transcriptase polymerase chain reaction (RT-PCR) method for genotyping RSV. Viral RNA was extracted from the NPA using guanidinium isothiocyanate method and purified with an RNAID commercial kit. Care-givers gave their consent prior to specimen collection. Administered questionnaires captured information on patient demographic and clinical history.

**Results:**

A total of 53 children were enrolled in the study with a male to female ratio of 3:1. Of the 53 NPA analyzed, 60.4% (32/53) were positive for RSV. Subsequent genotypic analysis showed that 72% (23/32) of the 60.4% RSV infections were RSV B only and 28% (9/32) were co-infections of both RSV A and B. Children between the ages of 2 - 12 months were the most affected age group per an RSV infection rate of 37.5% (12/32). No significant difference was detected in the recovery rate of ALRI (98.1%) and RSV (96.9%) positive patients from the infection. One patient died resulting in a mortality rate of 3.1%. Bronchopneumonia (20 out of 32 cases) was the major diagnosis on admission. RSV infection was seasonal dependent, described by 2 peaks in October and April-May.

**Conclusion:**

Both RSV A and RSV B genotypes co-circulated during the study period with RSV B predominating. RSV may possibly be the main pathogen of lower respiratory tract illness during epidemics in the wet seasons. Genotyping by the multiplex RT-PCR is one of the first attempts at molecular diagnosis of RSV infection in Ghana.

## Introduction

Acute lower respiratory tract infection (ALRI) is a leading cause of mortality in children less than five years worldwide [1, 2]. Studies have indicated that ALRI caused about 4 million child deaths each year particularly in infants (0-1 year) and two-third of deaths in preschool children [3, 4]. Recent study reviews have shown ALRI to be responsible for about 19% of all deaths in children less than five years; ninety percent of the deaths occurred in developing countries of which more than 70% took place in sub-Saharan Africa and South East Asia [5, 6]. Several reports have also documented ALRI as important cause of morbidity as well as a primary reason of hospitalization in children [7–10]. A number of agents including bacterial and viral etiologies have been associated with ALRI; however over 80% of acute respiratory infections have been attributed to respiratory viruses [1, 5, 11, 12]. The common respiratory viruses include respiratory syncytial virus (RSV), parainfluenzavirus, influenzavirus, rhinovirus and adenovirus. RSV causes proportionately more ALRI in infants, usually outranking all other microbial pathogens [13–16]. Thus RSV represents a substantial amount of burden of acute respiratory tract illness and is accountable for approximately 85% of cases of bronchiolitis and 20% of cases of childhood pneumonia mainly during the first year of life [9, 17–20]. Similar reports highlighted varied incidence and mortality of RSV from year to year in any one setting. [2, 21, 22]. Two serotypes of RSV, namely RSV A and RSV B, are known to exist with both of them exhibiting some evidence of heterotypic immunity and relatively similar clinical impact but children infected with RSV B appeared to have slightly more severe disease [23–27].

Considerable knowledge on RSV infections as a cause of yearly winter epidemics exists from the temperate regions whereas the problem has been studied to a lesser extent in the tropics [1, 10]. Studies from several industrialized countries acknowledged RSV as the main pathogen isolated in 19% of patients less than 5 years at the time of ALRI illness [9, 20, 28, 29]. The epidemiology of RSV in developing countries remains poorly defined. Nevertheless few denominator based studies that examine the role of RSV in causing severe lower respiratory infections have been acknowledged [15, 30, 31]. There is also an increasing recognition of the importance of RSV as cause of morbidity in the elderly [32].

Although reports have confirmed prevalence of RSV and ALRI worldwide, data from Ghana and sub-Saharan Africa is limited. The first study on ALRI in Ghana was in 1991 by Afari and colleagues at Gomoa Fetteh in the Central Region [33]. The study was based on clinical diagnosis and did not involve a laboratory confirmation of the etiological agents. Recent study has identified some bacterial and viral pathogens in children with ALRI but with scanty data on the circulating subtypes particularly for RSV [16]. Vigorous initiatives are being developed for the prevention and reduction of RSV disease worldwide. These among others include the development of vaccines for both childhood and maternal immunization [34–36]. Hence an initiation of RSV genotype surveillance is of high priority and importance to provide requisite baseline data as well as information for evaluating any future vaccines for RSV. Establishing RSV infection rates and seasonality is also very much needed to inform clinical care and management of ALRI in children. The study aimed to determine the prevalence of RSV and describe RSV genotypes circulation patterns in children with ALRI under 5 years of age in the Greater Accra Region.

## Methods

### Study setting and design

The study was carried out at the Child Health Department, Korle Bu Teaching Hospital. Korle-Bu Teaching Hospital is a major referral hospital located in Accra, the capital city of Ghana. The Child Health Department has an average yearly out-patient attendance of more than 36,000 [37]. During July to September, the peaks of the rainy season, about 3 children with clinical symptoms of ALRI are admitted daily (B. Goka, Personal communication).

### Study design

Based on average of 10% infection rate reported in children with ALRI from sub-Saharan Africa [15, 16, 30], a random convenient sampling was used to recruit study participants. The admissions & discharge (A&D) book at the Emergency Room of the Child Health Department was monitored on daily basis by trained personnel for eligible patients as per the eligibility criteria set for ALRI for children less than 5 years. Known asthmatics and children with abnormal cardiovascular systems were not enrolled in the study. The admitting physician at the Child Health Department managed all children recruited according to the standard departmental protocol for ALRI.

### Sampling and specimen collection

Children under 5 years with ALRI who sought medical assistance at the Department of Child Health were recruited into the study per their eligibility and with informed consent of parents or guardians. ALRI was defined as follows: Children <2 months having a breathing rate of equal to or more than 60 per minute; Children 2-11 months having a breathing rate of equal to or more than 50 per minute and Children 12 - 59 months having a breathing rate of equal to or more than 40 per minute; In addition to chest in-drawing or stridor or wheezing or apnea [38]. Respiratory specimens were obtained using the sterile single-use Argyle^®^ Delee Suction catheter with mucus trap kit. Initially nasopharyngeal aspirates (NPA) were collected by aspiration through a catheter and then washed down into the attached tube with 2ml sterile saline solution as transport medium. From February to November 2006, a total of 53 NPA were collected (one per each case enrolled). All specimens were kept on ice and transported immediately to the Department of Microbiology, University of Ghana Medical School where they were stored at -70°C in small aliquots until ready to be analyzed in the laboratory.

### Multiplex PCR and RSV Genotyping

From a total of 500µl NPA, viral nucleic acids were extracted and purified using guanidiniumisothiocyanate and RNaid kit (Q BIO gene, UK), according to the manufacturer′s instructions. cDNA was synthesized using random primers (20mU; PdN6; Pharmacia Biotech), and avian myeloblastosis (AMV) reverse transcriptase (Promega, USA), Amplification and detection of viral RNA was performed as previously described [39], but with slight modification on the Perkin Elmer GenAmp 2700 PCR Machine (Applied Biosystems, Ca, USA). The assay is a two-step PCR which first detected RSV in the primary PCR and then distinguished RSV subtype A from RSV subtype B in the nested PCR. The amplified products were separated on a 2% agarose gel stained with 0.5µg/ml ethidium bromide.

### Ethical approval

The study protocol was approved by the University of Ghana Medical School Ethical and Protocol Review Committee (UGMS E&PRC), College of Health Sciences, University of Ghana.

### Data management and analysis

Data was recorded and analyzed on EpiInfo version 3.3.2 statistical software for epidemiological data analysis. Statistical analyses were performed with SPSS17.0 software. Significance of differences in frequencies of various demographic and clinical features between various groups was tested using chi-square test and student's t-test. A p value of < 0.05 was considered to be statistically significant.

## Results

### Descriptive epidemiology

From February to November 2006, a total of fifty-three nasopharyngeal aspirates were collected from study subjects all of whom were children less than 5 years of age. The mean age was 13.4 months (range 2 weeks to 54 months). Most children, 42% (22/53) were in the 2 to 11 months age group. Male to female ratio of 3.3:1 and 2.6:1 was observed for ALRI and RSV infections respectively (p-value = 0.452). Majority of patients, 60.4% (32/53) were positive for RSV (Table 1, Table 2).

**Table 1:** Baseline demographics of ALRI and RSV-infected children at admission; values are numbers (%) unless otherwise stated

FACTOR	DETAILS	ALRI n = 53	RSV positives n = 32	P-value
Particulars of Subject	Gender (boys: girls)	40:13	23:9	0.452
	Mean Age in months	13.4 (63.2)	14.2 (62)	
Past illness and immunity	Similar illness in the past	20 (37.7)	14 (43.8)	0.005 0.389
	Exclusive breastfeeding up to 6 months	34 (64.2)	22 (68.8)	
	Median (range) days with breathing problems before admission	4 (0-30)	4 (2-30)	
Self-medication	Patients received antibiotics before admission	45 (84.9)	29 (90.6)	0.151
	Patients received antimalarial before admission	17 (32.1)	7 (21.9)	0.446
Outcome of disease	Median (range) duration (days) on admission	5 (0-33)	4 (0-32)	0.413
	Recovery rate	52 (98.1)	31 (96.9)	
	Mortality rate	1 (1.9)	1 (3.1)	

**Table 2:** Age distribution and detection rate of respiratory syncytial virus (RSV) infections in young children at the Korlebu Teaching Hospital

Age	No. of ALRI patients	RSV positive individuals No. (%)	P-value
0 – 2	8	6 (19)	0.49
2 – 11	22	12 (37.5)	
12 – 23	12	8 (25)	
24 – 35	6	2 (6.3)	
36 – 47	3	2 (6.3)	
48 – 59	2	2 (6.3)	
**0 – 59 (Total)**	**53**	**32 (60.4)**	

### Clinical outcome

**Figure 1:**
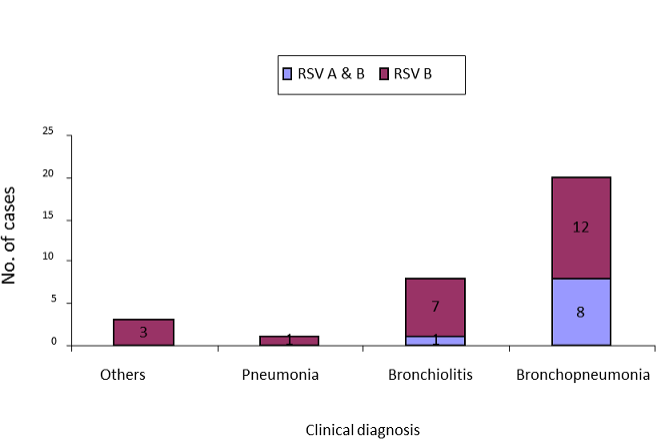
Most common admitting diagnosis and RSV genotypes

**Figure 2:**
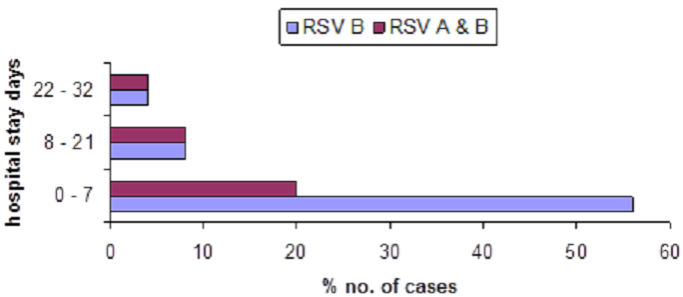
Duration of stay in hospital of children infected with the different genotypes of RSV

RSV infections were detected in all age groups with the highest rate in children from 0 – 23 months age group. However, no statistical difference (*p-*value = 0.49) was observed in infection rate among the age groups (Table 2). Almost all patients had a similar illness in the past or had received some form of medication such as antibiotics or antimalarial before admission to the hospital. Hundred percent of patients received antibiotics in addition to IV fluid and oxygen therapy while on admission (Table 1). The most common diagnosis of RSV infections as shown in Figure 1 were bronchopneumonia (62.5%), bronchiolitis(25%), pneumonia(3.1%) and respiratory distress (9.3%). The median hospital stay was 4 days (range 0 to 32 days) (Figure 2). The recovery rate reported among patients was 98.1% and 96.9% for ALRI and RSV respectively with no statistical difference. However, analysis of our results on similar illnesses in the past showed significant difference (p-value = 0.005) between ALRI and RSV (Table 1).

### Genotype analysis and distribution pattern

**Figure 3:**
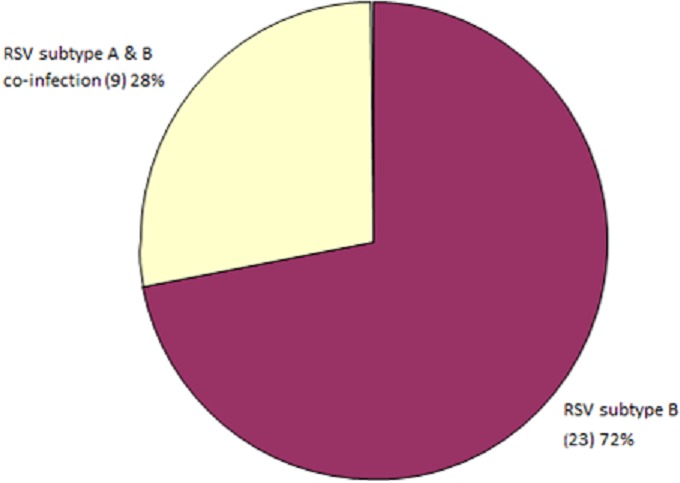
Prevalence of RSV genotypes A and B in children with ALRI

**Figure 4:**
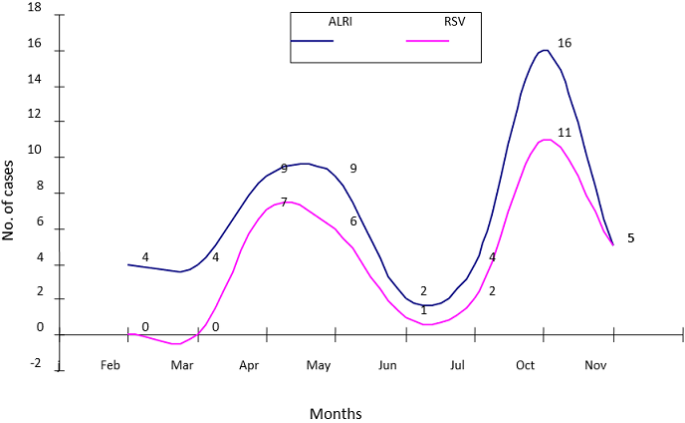
Monthly distributions of ARLI and RSV cases from February to November 2006

A total of 32 RSV isolates were identified and characterized during the study period. Analysis showed that both RSV genotypes A and B co-circulated during the study period. RSVB occurred predominantly with 72% (23/32) of samples; while RSVA was detected in 28% (9/32) of samples and in co-infection with RSVB (Figure 3). RSV infection was characterized by 2 peaks; more expressed peak was in October with 34% of cases and less expressed peak was in April and May with 22% and 19% of cases respectively. No RSV cases were observed in the month of February and March which are part of the dry season (Figure 4).

## Discussion

The study describes the prevalence and molecular analysis of RSV genotype detected during February to November 2006 in ALRI patients in Accra, Ghana. ALRI due to RSV infection in children determined by this study was 60.4%. Of these, 59.4% were in their first year of life. However, the rate decreased among children 3 years and older. These findings, compared to reports from other developing countries suggest that the prevalence of RSV infection observed in this study was higher [13, 14]. There were more males (40.6%) than females (18.8%) in all age groups. Other reports had similar observation, however it is not clear why this is so [15, 16]. Bronchopneumonia and bronchiolitis were the most severe complications associated with RSV infections. Treatment was largely supportive with oxygen and IV fluid therapy, but often ALRI patients were administered with antibiotics and or antimalarial. Antibiotic therapy was frequent among RSV infections while on admission, thus depicting RSV infection as a leading cause of antibiotic prescription. These findings also point out that ALRI are generally subjective to presumptive treatment and their causes are rarely sought. Nevertheless clinical presentations of ALRI are similar, therefore establishing the relative contribution to disease of individual pathogen is essential to steps leading to patient management and control.

Long duration stay at the hospital was a major burden on the hospital facilities such as bed turnover, staff and high expenditure on maintaining patients per day. This places a negative impact on public health and also stress and strain on the financial resources on the families involved; as caregivers had to absent themselves from work and home. Both group A and B RSV were found to be co-circulating during the study period, with RSVB being usually the predominant type with fewer dual infections. This observation is the first ever to be reported in Ghana. Similar trends had been suggested by studies within some European communities and developing countries [26, 27]. Genotyping using the multiplex RT-PCR is one of the first attempts at molecular diagnosis of RSV infection in Ghana. This assay was able to detect and confirmed the presence of RSV and thus useful in assessing the contribution of RSV to the overall burden of respiratory illness in the community and in diagnostic settings where subtype information might be sought.

RSV prevalence was seasonal dependent and coincided with the rainy seasons. The infections showed patterns with peaks occurring in October and April-May when epidemics generally start in the tropics. There were fewer cases of ALRI with no RSV isolated during part of the dry seasons, i.e. February and March. RSV infections have a characteristically regular seasonality both in the temperate and tropical climates [21, 40]. The high prevalence of RSV in childhood provides further support that, RSV vaccination may offer considerable public health benefit and provide effective prevention of RSV-associated ALRI in this population. This might in turn reduce all clinical ALRI admissions to the hospital by a half. While the development of a vaccine for target age group of early infants has focused on live virus vaccines, none has yet achieved requisite levels of both safety and immunogenicity [33, 34].

## Conclusion

The present study identified 60% of RSV infections in ALRI children in a hospital setting in Accra, Ghana. Both RSVA and RSVB genotypes co - circulated during the study period with RSVB predominating. RSV infection was seasonal dependent so during epidemics in the wet seasons, RSV may possibly be the main pathogen of lower respiratory tract illness among young children in Ghana. Genotyping by the multiplex RT-PCR is one of the first attempts at molecular diagnosis of RSV infection in Ghana and therefore provides insights into the epidemiology and allows comparative analyses of worldwide circulating RSV.
